# Influence of Jump and Ball Release Parameters on Shooting Accuracy in Basketball Under Varying Constraints

**DOI:** 10.3390/jfmk10040459

**Published:** 2025-11-21

**Authors:** Catarina M. Amaro, Maria António Castro, Rui Mendes, Hannah Rice, Beatriz B. Gomes

**Affiliations:** 1 University of Coimbra, CIPER (Interdisciplinary Center for the Study of Human Performance), Faculty of Sport Sciences and Physical Education, 3040-248 Coimbra, Portugal; catarinammamaro@gmail.com; 2 University of Coimbra, CEMMPRE (Centre for Mechanical Engineering, Materials and Processes), 3030-788 Coimbra, Portugal; maria.castro@ipleiria.pt; 3 School of Health Sciences, CiTechCare, CDRSP, Polytechnic University of Leiria, 2411-901 Leiria, Portugal; 4 Polytechnic Institute of Coimbra, Coimbra Education School, 3030-329 Coimbra, Portugal; rmendes@esec.pt; 5 SPRINT Sport Physical Activity and Health Research & Innovation Center, 3030-329 Coimbra, Portugal; 6 Department of Physical Performance, Norwegian School of Sport Sciences, Sognsveien 220, 0863 Oslo, Norway; hannahr@nih.no

**Keywords:** sports biomechanics, shooting accuracy, opposition, gym audience noise, ball kinematics, jump shot

## Abstract

**Background**: This study investigates how both jump-related (jump height and flight time) and ball-related parameters (release height, release angle, and velocity) influence shooting accuracy in basketball under different contextual constraints. **Methods**: Eighteen senior players competing in the national championship (11 females and 7 males; 22.0 ± 3.7 years) performed 90 shots each across three positions (left 45°, middle 90°, right 45°) and three shooting conditions (baseline, simulated gym audience noise, and simulated opposition). Jump variables were derived from force platforms, while ball kinematics were extracted using a high-speed Qualisys camera system. **Results**: A three-way ANOVA revealed no systematic effects of position or opposition, and only a small effect of noise on flight time (*p* = 0.019), which was not confirmed by the Linear Mixed Model. Comparisons between successful and missed shots indicated significantly higher flight time, jump height, and release height, and a tendency for higher release velocity in successful attempts, with no differences in release angle. Spearman correlation showed weak associations between biomechanical variables and shooting accuracy (R^2^ = 0.005–0.012). **Conclusions**: These findings suggest that while adaptive biomechanical changes occur under contextual constraints, their isolated impact on shot success is limited. Successful performance appears to rely more strongly on release-related parameters, emphasizing the need for a holistic approach to training that integrates technical, perceptual, and psychological dimensions.

## 1. Introduction

Shooting is one of basketball’s most important technical skills, playing a central role in a team’s offensive success. The accuracy and effectiveness of a shot depends on various biomechanical factors, such as jump height, flight time, and arm-leg coordination, all of which influence the ball’s trajectory and the shooter’s ability to overcome defenders [1]. Understanding these variables across different shooting conditions and how they impact the accuracy of shots taken by experienced athletes is essential for developing effective training protocols and strategies.

Research has shown that the mechanics of a basketball shot are influenced by various factors, including player fatigue, psychological stress, and physical conditioning [2,3]. For instance, Erculj and Supej (2009) demonstrated that player fatigue negatively impacts shot accuracy, highlighting the importance of endurance training for basketball players [2]. Similarly, psychological stress, such as the presence of a noisy crowd, has been found to affect shooting performance [4,5]. Oudejans et al. (2011) investigated the impact of audience noise on free-throw shooting and found that noise can increase anxiety levels, leading to decreased performance [4]. Recent findings emphasize the role of mental focus and the ability to maintain technique consistency under pressure [6].

Previous studies have shown that external stressors, such as audience noise and defensive pressure, can affect athletes’ focus, decision-making, and motor execution, ultimately influencing shooting accuracy. Some authors demonstrated that audience noise can increase cognitive anxiety and disrupt concentration during skill execution [7]. Similarly, Baumeister (1984) highlighted the phenomenon of “choking under pressure,” where heightened stress leads to impaired motor performance [8]. Defensive pressure has also been shown to impact visual focus and shooting mechanics, as noted in studies of quiet eye duration [9]. These factors simulate real-game environments, providing a more ecologically valid context for understanding how athletes adapt under pressure. Incorporating such conditions into the study not only enhances its practical relevance but also bridges the gap between controlled experiments and real-world competition scenarios.

Physical conditioning and training methods also play a significant role in shooting accuracy. Studies have indicated that specific strength and conditioning programs can improve jump height and shooting performance [10]. Additionally, a study emphasized the role of muscle coordination in achieving optimal jump height and flight time, further supporting the need for targeted training programs [11].

Jump height is a crucial aspect of the basketball jump shot as it can influence the shooting angle and the trajectory of the ball, thus affecting the likelihood of a successful shot. Research by Okazaki et al. (2015) has demonstrated that greater jump height can provide players with a better vantage point to release the ball, potentially avoiding defenders and increasing the chance of scoring [1]. Similarly, Inaba (2019) emphasized that optimal jump height allows for a more controlled and accurate shot release [12]. Research on flight time and shooting performance in basketball and handball has yielded mixed results. While extended flight time during jump shots is often thought to improve stability and control, studies have found no clear correlation between countermovement jump ability and sport-specific jumping performance [13].

A combined plyometric and shooting training program improved vertical jump outcomes, including flight time, in young male basketball players [14]. The presence of a defender contesting shots led to changes in movement execution and gaze behavior, including longer jump time and ball flight time [15]. Interestingly, the impact on shooting accuracy varied among players, with some improving and others declining in contested situations [15]. These findings highlight the complex relationship between flight time, stability, and shooting performance in basketball and related sports [16].

Research on basketball jump shots reveals that defensive pressure significantly alters shooting mechanics. Players tend to increase release angles, reduce flight time, and make postural adjustments when facing opponents [17]. As obstacle height increases, players exhibit higher jumps and greater ball entry angles but shooting accuracy decreases [18]. Defensive pressure leads to kinematic adjustments, and these changes aim to release the ball more quickly and from a greater height to avoid interception. Contested shots result in shorter execution times, longer jump and ball flight times, and changes in gaze behavior [15]. Defensive pressure significantly influences shooting efficiency and game outcomes, with winning teams achieving better shooting percentages due to improved team cooperation and more open scoring opportunities [19]. These findings highlight the importance of incorporating representative constraints in training to prepare players for actual game situations better [15].

Beyond the commonly studied lower-body metrics such as jump height and flight time, recent research has emphasized the critical role of ball release (BR) parameters—namely, release height, release angle, and release velocity—in shaping shooting performance. These variables directly influence the ball’s flight path, affecting its arc, speed, and capacity to avoid defensive interference. Higher release points have been associated with an increased likelihood of successful shots, as they allow athletes to shoot over defenders more effectively. Studies noted that greater vertical displacement and higher release height may enhance scoring chances, especially in contested situations [20,21].

In addition, BR angle determines the trajectory and entry angle of the ball. Variations in shooting distance and body orientation can lead players to adapt their release angle to maintain optimal ball arc and scoring probability. Okazaki and Rodacki (2012) found that experienced players adjust their release angle in response to spatial constraints, ensuring a controlled and consistent shooting form [20]. Similarly, Rojas et al. (2000) reported that changes in release angle are part of a broader set of biomechanical adjustments made when players shoot under pressure [17].

Although release velocity has received comparatively less attention, it plays a crucial role in the successful execution of the shot. Caseiro et al. (2023) observed that skilled players increase ball speed when shooting from lateral positions or greater distances, likely to maintain the necessary momentum and precision [22]. Cowin (2022) further emphasized that elite performers demonstrate high motor adaptability, dynamically modulating release variables, including speed, to maintain accuracy despite varying environmental and tactical demands [23].

Taken together, these findings highlight the importance of analyzing not only lower-limb outputs but also ball release mechanics. A comprehensive understanding of shooting performance must therefore consider the interplay between release height, angle, and velocity, especially when athletes are exposed to situational constraints such as noise and defensive pressure. Incorporating these variables into biomechanical analyses enables a more holistic view of how players adapt their technique under realistic game-like conditions.

Shooting techniques across various sports are essential for understanding performance. In basketball, specific biomechanical variables, such as jump height and flight time, play a key role in shooting performance. Research has demonstrated that stability and a higher release point relative to standing height are associated with improved free throw performance. Additionally, the importance of the kinetic chain is often emphasized, suggesting that coordinated movement from the lower body through to the shooting arm is crucial for accuracy [24].

The advancement of technology in sports science has significantly enhanced the understanding of these performance-related variables. Methods such as force platforms allow precise measurement of key parameters, such as jump height and flight time. These technologies provide valuable insights into the specific biomechanical factors that influence shooting accuracy [25,26]. The widespread application of such technologies across major sports has transformed data collection, processing, and athlete monitoring in both training and competition environments [27].

The primary objective of this study was to assess whether differences exist in jump height, flight time, and BR parameters (BR height, BR angle, and BR velocity) during basketball shots taken from various positions (left 45°, 90°, and right 45°) and under different shooting conditions (baseline, simulated gym audience noise, and opposition) amongst competitive basketball players. Additionally, the study aimed to evaluate the relationship between these kinematic variables (jump height, flight time, and BR parameters) and shot accuracy. Based on existing evidence, it was hypothesized that shooting under opposition would result in adaptive changes, leading to increased jump height, extended flight time, and altered BR characteristics compared to shots taken under other conditions.

## 2. Materials and Methods

### 2.1. Participants

The study sample comprised 18 athletes (11 females and 7 males) from senior teams competing in national championships, with an average age of 22.0 years (± 3.70), height of 169.70 cm (± 9.59), and mass of 68.50 kg (± 13.29), performing 90 shots each. When divided by sex, the female players (*n* = 11) presented a mean age of 21.00 ± 3.43 years, height of 165.40 ± 4.72 cm, and body mass of 63.00 ± 7.63 kg, while the male players (*n* = 7) showed a mean age of 26.00 ± 3.62 years, height of 178.90 ± 7.40 cm, and body mass of 75.60 ± 14.40 kg. All participants had at least 7 years of experience in federated basketball (average 12.50 ± 4.23 years, 11.00 ± 4.10 years for females, 16.00 ± 4.32 years for the males) and reported no injuries in the three months preceding the study. The sample size of 18 athletes is reasonable and aligns with similar research involving basketball performance or biomechanical analysis [28].

Before participating, all athletes were fully briefed on the study’s objectives and procedures and provided written informed consent. The ethical committee of the Faculty of Sports Science and Physical Education at the University of Coimbra approved the study, which was conducted following the Declaration of Helsinki (approval number CE/FCDEF-UC/00812021). All data were collected and stored anonymously to ensure participant confidentiality.

### 2.2. Protocol

The protocol began with a 10 min warm-up, including shooting drills and functional exercises. After that, the athlete was asked to stand on the two force platforms and initiate all shots from this position. No instructions were provided regarding how or where to land, to avoid influencing the athlete’s normal movement, just to do the normal shot and try to score.

**Figure 1:**
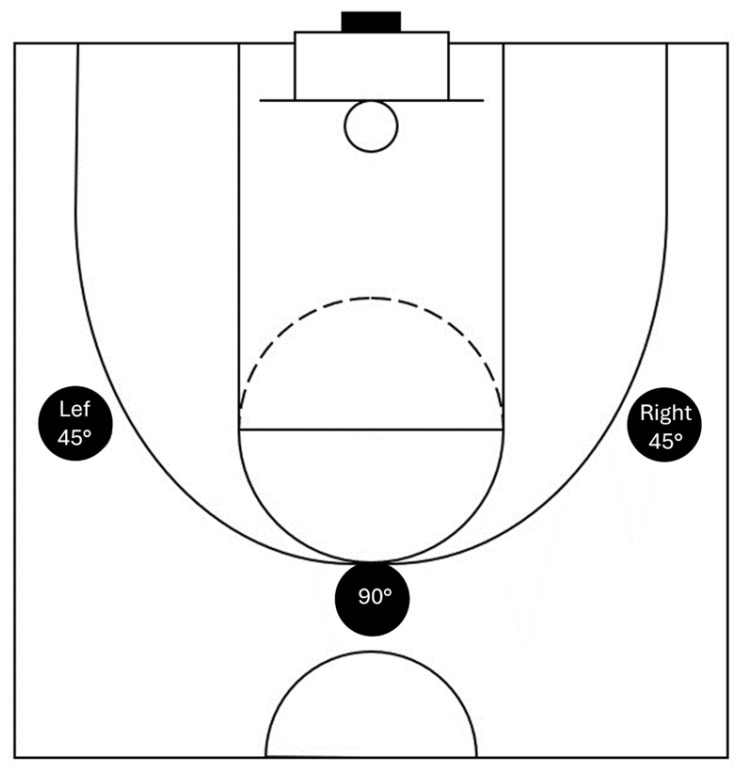
Shooting positions.

All athletes executed ten shots from three distinct shooting positions (right 45°, 90°, and left 45°), maintaining a consistent distance of 6.75 m from the board, (Figure 1) and under varying constraints (no constraints (B), simulated gym audience noise (N), and simulated opposition (O)). The shooting sequence was randomized to minimize the impact of factors such as learning effects and fatigue on performance. In basketball shooting, performance can vary due to external and internal factors (e.g., fatigue, focus). By randomizing the sequence, the likelihood of any single shot type consistently occurring at the end of the shooting sequence, where fatigue could be more pronounced, was minimized. Additionally, while the participants took 90 shots, this volume reflects typical training scenarios for basketball players, where repeated shooting is common. Given that the participants were skilled athletes accustomed to such physical demands, it is reasonable to expect that their endurance and capacity to maintain shooting performance were within normal ranges. By collecting 10 attempts in each condition, we average out inconsistencies and capture a representative mean performance for each condition. Research indicates that a minimum of 8–15 repetitions is often used for movement-based tasks to stabilize kinematic and kinetic data [1,29]. Athletes were given a 30 s rest period between conditions at the same position and a 2 min rest period between different positions.

Environmental constraints (simulated gym audience noise) and task constraints (simulated opposition) were incorporated to evaluate their impact on motor behavior during shooting. The opposition was simulated using adjustable equipment calibrated to different heights based on each athlete’s height (1.20× shooter’s height) and positioned at a constant distance of one meter from the shooter [30]. Simulated gym audience noise, set at 105 dBA [31], began before the athlete received the ball for their first shot under this condition. This noise, generated by using external speakers, remained constant across all ten shots, mimicking a hostile crowd environment. The same audio recording was used for all players and shots under this condition.

Data collection occurred in the same gymnasium, using a game ball inflated to the recommended pressure of 0.62 bar. The ball size varied between female and male players according to official regulations. All participants wore their standard basketball footwear and practice uniforms. Each data collection session lasted approximately 60 min.

### 2.3. Equipment

**Figure 2:**
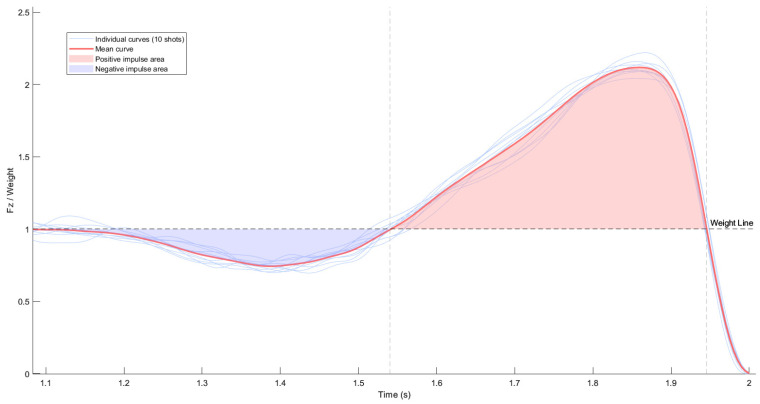
Fz during a jump shot normalized to body weight. Blue area below the body weight line = negative impulse; red area above the body weight line = positive impulse. Body weight is represented as a dashed horizontal line.

Kinetic data from each jump shot was collected using two Bertec force platforms (model 4060-05; Bertec Corporation, Columbus, OH, USA) at a sampling frequency of 1000 Hz. For each individual basketball shot, the vertical ground reaction force (Fz) was obtained by summing the vertical force signals from both platforms, thereby generating a single representative signal for subsequent analysis. Based on this combined Fz signal, the negative, positive, and net vertical impulse were calculated based on Fz obtained from a force platform, by using a custom MATLAB R2022b (MathWorks, Natick, MA, USA) routine. The athlete’s body mass (in kg) was entered manually and adjusted by +1.77 kg to account for equipment (e.g., shoes, tracking devices, clothing). The negative impulse (Figure 2) was defined as the area between Fz and body weight from the last point where Fz dropped below body weight until the beginning of the propulsive phase (i.e., when Fz exceeded body weight again). The positive impulse was computed from that point until the final crossing of the body weight line prior to take-off (Figure 2). The net impulse was calculated as the sum of both:

(1) $$A=I_{positive}+I_{negative}$$

where *I_positive_* and *I_negative_* are the positive and negative impulse, respectively.

The vertical take-off velocity of the center of mass was estimated using the impulse–momentum relationship:

(2) $$v_{0}=\frac{I_{net}}{m}$$

where *v*_0_ is the vertical take-off velocity (m/s), *I_net_* is the net vertical impulse (N ∗ s), and *m* is the body mass (kg).

Based on this velocity, the jump height and flight time were calculated as follows:

(3) $$h=\frac{{v_{0}}^{2}}{2g};\;t_{flight}=\frac{{2v}_{0}}{g}$$

where *h* is the jump height (m), *v*_0_ is the vertical take-off velocity (m/s), *g* is the gravitational acceleration (9.81 m/s^2^), and *t* is the flight time (s).

For each individual basketball shot, all calculations were based on raw, unnormalized data. Figure 2 illustrates an example of the vertical ground reaction force curve normalized to body weight for 10 shots in one shooting condition of one athlete, and a mean curve with the negative and positive impulse areas clearly delimited relative to body weight line.

**Figure 3:**
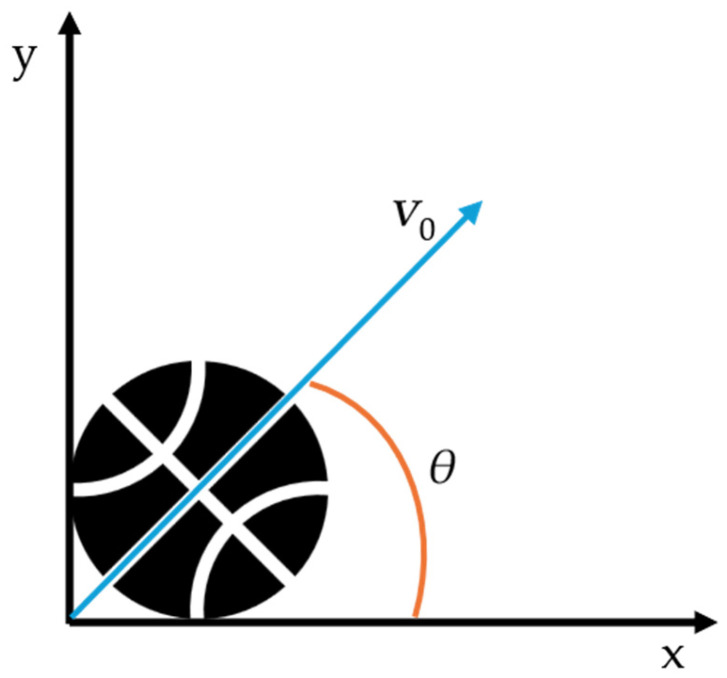
Schematic representation of the release angle (*θ*) in basketball shooting. The angle is defined between the horizontal axis (x) and the initial velocity vector of the ball (*v*_0_) at the moment of release.

Ball kinematic data were obtained using a Qualisys motion capture system (Qualisys AB, Gothenburg, Sweden) composed of high-speed infrared cameras operating at 200 Hz. Four thin flat reflective adhesives were attached symmetrically to the surface of each basketball to define a local coordinate system and allow accurate 3D tracking of the ball’s motion. The release moment was identified based on the peak vertical acceleration of the ball, determined by the mean of the visible markers, and confirmed via visual inspection in Qualisys Track Manager (QTM, 2023.3; Qualisys AB, Gothenburg, Sweden). The release angle (*θ*) was defined as the angle between the horizontal axis and the initial velocity vector of the ball (*v*_0_) at the moment of release, as illustrated in Figure 3. The motion capture system was temporally synchronized with the force platforms, allowing precise alignment of the ball kinematic data with ground reaction force data for each trial.

Shooting accuracy was assessed using a scoring system ranging from 0 to 4: 0 was given if the shot missed without touching the board or the hoop; 1 was given if the shot missed touching the board and/or the hoop; 2 was given if the shot scored with the ball touching the board; 3 was given if the shot scored with touching the hoop; and 4 was given if the shot was converted without the ball touching the board or the hoop [32]. The athletes were not informed of the scoring criteria before data collection.

### 2.4. Statistics Analysis

The data were analyzed using a personalized MATLAB routine to calculate jump height and flight time during the shot, and IBM SPSS Statistics 26.0 (IBM Corporation, New York, NY, USA) [33]. Descriptive statistics for the entire sample were reported as mean ± standard error with 95% confidence intervals. As an omnibus screening analysis, a three-way ANOVA (General Linear Model—Univariate) with fixed factors Position (3 levels), Noise (2 levels), and Opposition (2 levels) was first performed for each dependent variable (jump height, flight time, BR height, BR angle, and BR velocity). Partial eta squared (η^2^*p*) was reported as an effect size. Because the protocol did not include trials with simultaneous noise and opposition, the Noise × Opposition and Position × Noise × Opposition interactions were not estimable. Repeated measures ANOVA was employed for comparisons between shooting conditions and angles (nine conditions in total). A Bonferroni adjustment was applied to control for Type I errors.

In addition, Linear Mixed Models (LMMs) were used to account for the repeated-measures structure of the data. For this purpose, flight time was entered as the dependent variable, condition (noise vs. no noise, with no noise defined as baseline + opposition) as a fixed factor, and athlete as a random intercept. Estimated marginal means (EMMs) were calculated, and pairwise comparisons were performed with Bonferroni adjustment. This approach allowed for the assessment of potential differences in flight time between conditions, while controlling for within-subject variability [34].

Given that the outcome variable (scored vs. missed) is dichotomous and the predictors (e.g., BR height, velocity, angle) are continuous but not guaranteed to follow a normal distribution, this nonparametric test was deemed appropriate. This methodological approach has been previously used in basketball performance research to assess differences in shooting mechanics under binary scoring outcomes [1,35]. For example, Okazaki et al. compared kinematic differences between made and missed shots under varying levels of defensive pressure using similar nonparametric statistics, highlighting that subtle biomechanical differences can meaningfully influence shot success. Furthermore, Mann–Whitney U is recommended when analyzing skewed or non-normally distributed sports data, especially in skill-based tasks where performance variability is high [2,17]. Therefore, applying this test allowed us to assess whether specific release parameters differed significantly between shots that were scored and those that were not, without relying on assumptions of normality or equal variances.

Additionally, to explore associations between the ordinal shooting accuracy score (0–4) and biomechanical variables, Spearman’s rank correlation coefficient (ρ) was computed. Therefore, non-parametric correlations were used to examine monotonic relationships between accuracy and jump or ball-release parameters (height, angle, and velocity).

## 3. Results

From the total of 1620 shots initially recorded (18 players × 10 shots × 3 positions × 3 conditions), only 1553 were included in the final analysis of force data. The remaining 67 shots (4.1%) were excluded due to technical errors in data collection, such as incomplete or corrupted recordings, or because the shots did not meet the experimental protocol. These criteria were defined before data collection to ensure the consistency and reliability of the dataset. Regarding ball kinematic, from the original data, only 710 were retained for analysis due to tracking issues with the Qualisys system, such as marker occlusion or loss of visibility during the release. From the total of 1553 valid jump trials obtained from the force platforms, only 710 also contained complete ball kinematic data due to occasional marker occlusion or temporary loss of visibility during high-speed ball motion in the Qualisys system. The Qualisys and Bertec systems were temporally synchronized, and these 710 trials therefore represent a subset of the full force dataset for which both kinetic and kinematic information were simultaneously available. All analyses linking jump-related and BR variables were conducted exclusively on this synchronized subset to ensure that the variables originated from the same trials. Although this reduction in sample size may slightly limit the external validity of the kinematic results, it preserves the internal validity of all cross-variable analyses by ensuring strict data correspondence across measurement systems. These limitations, common in optical motion capture of fast-moving objects, led to the exclusion of trials that did not meet predefined quality criteria.

Table 1 presents the descriptive statistics (mean ± standard deviation) for all variables—jump height [m], flight time [s], BR height [m], BR angle [°], BR Velocity [m/s] and accuracy, across the different shooting conditions and positions.

As shown in Table 1, mean values of jump height and flight time were consistent across shooting conditions and positions, with limited variation between trials. Ball release height ranged approximately from 2.02 to 2.13 m, while release angle values were between 54.5° and 57.9°. Release velocity remained stable across conditions, with averages close to 6.7–7.0 m·s^−1^. Accuracy scores showed greater variability, ranging from 1.4 ± 1.3 to 2.1 ± 1.4 across conditions.

Table 2 presents the results of three-way ANOVA.

The three-way ANOVA revealed no significant main effects of position (F(2,34) = 0.82, *p* = 0.449, η^2^*p* = 0.002), noise (F(1,17) = 2.99, *p* = 0.092, η^2^*p* = 0.004), or opposition (F(1,17) = 0.27, *p* = 0.606, η^2^*p* = 0.000) on jump height. Similarly, for flight time, no significant effects were found for position (F(2,34) = 1.88, *p* = 0.171, η^2^*p* = 0.005) or opposition (F(1,17) = 0.16, *p* = 0.694, η^2^*p* = 0.000), while a small but statistically significant effect of noise was observed (F(1,17) = 6.38, *p* = 0.019, η^2^*p* = 0.008). For all ball release parameters (height, angle, and velocity), none of the main effects or interactions reached statistical significance (all F(1–2,17–34) ≤ 2.49, *p* ≥ 0.13, η^2^*p* ≤ 0.005). According to conventional benchmarks for partial eta squared (η^2^*p*), values below 0.01 are considered small, 0.01–0.06 moderate, and above 0.14 large [34]. The η^2^*p* values observed in this study were all below 0.01, indicating that the practical magnitude of the effects was small, even when statistically significant. This indicates that players generally maintained stable release and jump mechanics across conditions.

**Figure 4:**
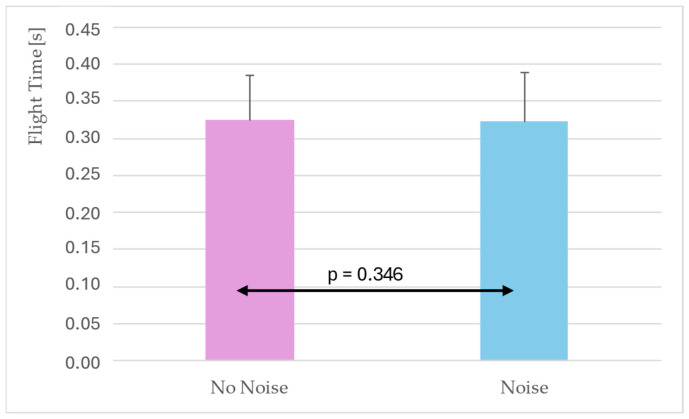
Comparison of flight time between no-noise (baseline + opposition) and noise conditions. Bars represent estimated marginal means ± standard error. Significance level *p* < 0.05.

To further examine the effect of noise on flight time, conditions were grouped into “no noise” (baseline and opposition) and “noise.” This contrast was intended to determine whether the presence of simulated crowd noise systematically altered flight time in the jump shot (Figure 4).

Flight time did not differ significantly between noise (0.322 ± 0.067 s) and no-noise (0.325 ± 0.061 s) conditions (*p* = 0.346) (Figure 4). The mean difference was minimal (≈ +0.003 s), corresponding to a non-significant increase of 0.9% under noise.

**Figure 5:**
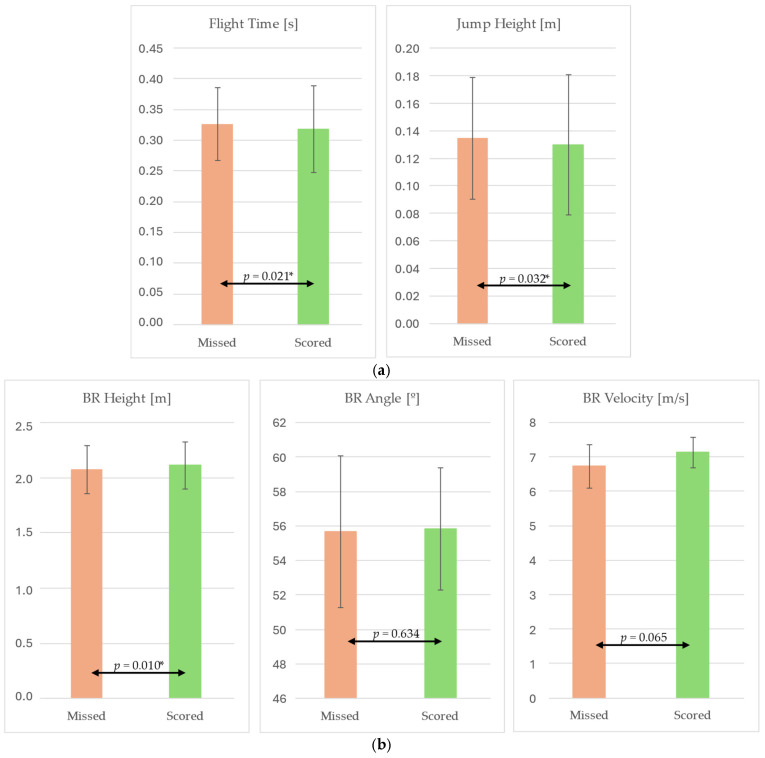
Comparison of (**a**) jump height and flight time, and (**b**) ball release variables between successful and missed shots (independent-samples *t*-test). Significance level of *p* < 0.05 *.

Significant differences were observed between successful (scored) and unsuccessful (missed) shots in flight time and jump height, with lower values recorded in scored attempts (Figure 5).

At ball release, successful shots also presented a significantly higher release height compared to missed attempts. No significant differences were found for release angle, while release velocity showed a trend towards higher values in successful shots.

To examine the relationship between biomechanical variables and shooting accuracy on the 0–4 ordinal scale, Spearman’s rank correlation coefficients (ρ) were calculated (Table 3).

Weak but statistically significant positive correlations were observed between ball-release height and shooting accuracy (ρ = 0.116, *p* = 0.002) and between ball-release velocity and accuracy (ρ = 0.084, *p* = 0.025). In contrast, flight time and jump height correlated weakly and negatively with accuracy, while release angle showed no significant association.

## 4. Discussion

The omnibus three-way ANOVA indicated that neither shooting position nor simulated opposition systematically affected jump- or BR-related variables, while simulated noise showed a small but statistically significant effect on flight time. When this factor was examined more closely with a Linear Mixed Model accounting for repeated measures at the individual level, the apparent effect was reduced to a minimal increase of approximately +0.003 s (~0.9%) and was not statistically significant (*p* = 0.346). Overall, these results suggest that experienced athletes maintain stable shooting mechanics even when exposed to contextual constraints such as simulated noise, reinforcing the concept of motor robustness in skilled performers [1,23]. The small effect sizes (η^2^*p* < 0.01) observed across conditions further support the notion that these contextual constraints had minimal practical impact on players’ mechanics. Such small magnitudes indicate that while differences may reach statistical significance, their real-world influence on performance is likely negligible.

This stability is consistent with studies indicating that elite basketball players are able to maintain consistent release kinematics across changing contexts by relying on finely tuned adjustments rather than large biomechanical modifications [1,23]. The minimal impact of noise in our study also parallels findings by van Maarseveen and Oudejans (2018), who showed that defensive pressure influences movement execution but does not necessarily enhance accuracy [15]. Similarly, Kambič et al. (2022) found that opponent height can trigger biomechanical adjustments not easily replicated under controlled experimental designs, suggesting that while simulated constraints may not fully capture the pressures of competition, they provide insight into the resilience of shooting mechanics under task demands [18].

When comparing successful and missed shots, clear biomechanical differences emerged. Significant differences were observed in flight time, jump height, and BR height, with successful attempts consistently presenting higher values in ball parameters, but lower values in jump height and flight time. These findings suggest that successful shots are facilitated by a higher release point, which improve clearance and trajectory conditions [1,21]. BR velocity also showed a tendency to be higher in successful attempts, which is consistent with literature linking faster release speeds to improved trajectory control and precision, especially under time pressure or defensive constraints [23]. Interestingly, BR angle did not differ between scored and missed shots, echoing Miller and Bartlett (1996), who argued that while release angle contributes to arc control, it may not be as critical as release height or velocity when other kinematic factors are optimized [21].

These outcome-based comparisons emphasize the value of analyzing shooting not only across experimental conditions but also in terms of success versus failure, as they highlight localized adaptations that may not be evident in global analyses. Our findings align with previous work demonstrating that outcome-based approaches can reveal the biomechanical features most directly associated with shot success [1,23].

Spearman correlation analyses confirmed that the relationships between biomechanical variables and accuracy were weak, reinforcing the multifactorial nature of shooting performance. Although statistically significant associations were found, the relationships were weak: flight time and jump height correlated negatively with accuracy, while BR height and BR velocity correlated positively. This suggests that while biomechanical parameters provide necessary conditions for effective shooting, they are far from sufficient to explain success in isolation. This reflects the multifactorial nature of performance, where perceptual, tactical, and psychological factors also play essential roles. Shooting performance is likely shaped by a complex interplay of perceptual, psychological, and tactical influences, consistent with the multidimensional framework described by Joseph et al. (2021) [35].

This interpretation is further supported by Boddington et al. (2020), who noted that basketball shooting accuracy tests may be subject to floor and ceiling effects, limiting their sensitivity to subtle biomechanical variations [36]. Accordingly, our findings highlight that while jump height and flight time are important performance markers, they contribute only indirectly to accuracy, which depends more heavily on upper-limb release mechanics and situational context. Teramoto and Cross (2017) also emphasized that height exerts a stronger influence at the tactical and collective level of play rather than through individual shot mechanics, further underscoring that jump-related variables are only part of a broader performance puzzle [37]. Similarly, Erčulj and Supej (2018) showed that fatigue significantly alters jump shot dynamics, pointing to the relevance of external factors not included in the present design [38].

Taken together, the results of this study highlight the robustness of shooting mechanics in experienced players under varying constraints, while also identifying key biomechanical factors that distinguish successful from unsuccessful attempts. The weak but significant correlations with accuracy suggest that while biomechanical adjustments provide a foundation, accuracy is ultimately determined by the integration of technical, perceptual, and psychological elements. These insights align with the concept of motor adaptability and reinforce the importance of adopting a holistic view of performance, where both upper- and lower-body mechanics, along with environmental and psychological factors, are considered [21,23].

From a practical standpoint, these findings suggest that training should emphasize the refinement of release mechanics—particularly release height and velocity—while also incorporating environmental and task constraints such as noise and defensive pressure. Although the immediate biomechanical effects of these constraints appear modest, they can prepare athletes for the perceptual and psychological demands of competition. Coaches should design drills that replicate competitive conditions, encouraging athletes to maintain consistency in shot mechanics under pressure, while simultaneously reinforcing decision-making and tactical awareness.

This study has several limitations. Firstly, the relatively small and homogeneous sample may limit generalizability to broader populations or different competitive levels. Secondly, the controlled environment of the study, while valuable for isolating biomechanical effects, may not fully reflect the dynamics of real-game contexts, where tactical interactions and psychological pressure strongly influence performance. Another limitation concerns the loss of ball-tracking data. Importantly, the missing data were distributed randomly across athletes and shooting conditions, with no systematic pattern observed between the baseline, noise, or opposition scenarios. Therefore, although this reduction in usable trials decreased the total sample size for ball-related analyses, it is unlikely to have introduced bias affecting the direction or interpretation of the results. Finally, the scope of variables was limited, excluding other kinetic, perceptual, and cognitive measures that could provide further insights into performance determinants.

Future research should build on these findings by employing larger and more diverse samples, simulating more realistic game conditions, and including additional biomechanical and perceptual variables. The hypothesis proposed in the introduction assumed that contextual constraints such as opposition and noise would induce adaptive changes in jump height, flight time, and ball release parameters. Our results, however, indicate that these effects were minimal: opposition showed no systematic influence, and the small effect of noise on flight time detected in the omnibus ANOVA was not confirmed by the Linear Mixed Model. Moreover, these variations did not translate into improvements in shooting accuracy, suggesting that while adaptive biomechanical adjustments occur, their direct impact on shot success is limited.

## 5. Conclusions

This study showed that although simulated constraints such as noise and opposition produced minor adjustments in jump height, flight time, and BR parameters, their isolated effect on shooting accuracy was negligible. The small effect detected only of noise on flight time by ANOVA reinforce the stability of shooting mechanics in experienced players. Comparisons between successful and missed shots revealed that higher flight time, jump height, and BR height, as well as a trend towards higher BR velocity characterized successful attempts. Correlation analyses demonstrated only weak associations between biomechanical variables and shooting accuracy, indicating that scoring success depends on a multifactorial interaction of technical, perceptual, and psychological factors.

Practically, these findings suggest that training should focus on refining release mechanics—particularly height and velocity—while incorporating representative constraints to strengthen resilience and adaptability under game-like conditions.

## Figures and Tables

**Table 1:** Descriptive statistics (mean ± standard deviation) for jump variables (jump height and flight time), BR variables (height, angle and velocity) and accuracy for the sample (*n* = 18).

Shooting Conditions	Mean ± SD					
	Jump Height [m]	Flight Time [s]	BR Height [m]	BR Angle [°]	BR Velocity [m/s]	Accuracy [0; 4]
Baseline Right	0.25 ± 0.08	0.44 ± 0.07	2.13 ± 0.13	54.45 ± 3.23	6.99 ± 0.58	1.7 ± 1.4
Baseline Middle	0.23 ± 0.09	0.42 ± 0.08	2.09 ± 0.14	56.91 ± 2.48	6.78 ± 0.56	2.0 ± 1.5
Baseline Left	0.25 ± 0.09	0.44 ± 0.08	2.02 ± 0.20	57.47 ± 2.73	6.72 ± 0.63	1.7 ± 1.4
Noise Right	0.25 ± 0.08	0.44 ± 0.07	2.09 ± 0.20	54.79 ± 3.81	6.90 ± 0.70	1.7 ± 1.4
Noise Middle	0.24 ± 0.08	0.44 ± 0.08	2.11 ± 0.16	55.78 ± 2.84	6.79 ± 0.76	2.0 ± 1.5
Noise Left	0.25 ± 0.08	0.44 ± 0.07	2.07 ± 0.15	56.68 ± 3.31	6.80 ± 0.74	2.0 ± 1.5
Opposition Right	0.25 ± 0.08	0.43 ± 0.10	2.10 ± 0.15	55.17 ± 3.27	6.95 ± 0.73	1.6 ± 1.5
Opposition Middle	0.25 ± 0.09	0.44 ± 0.08	2.11 ± 0.14	55.40 ± 4.46	6.90 ± 0.74	1.7 ± 1.4
Opposition Left	0.25 ± 0.09	0.44 ± 0.08	2.10 ± 0.14	57.20 ± 2.46	6.85 ± 0.71	1.4 ± 1.3

**Table 2:** Results of the three-way ANOVA (Position × Noise × Opposition) for jump-and BR related parameters.

Effect	*p*, η^2^*p*				
	Jump Height	Flight Time	BR Height	BR Angle	BR Velocity
Position	0.449, 0.002	0.171, 0.005	0.437, 0.002	0.510, 0.002	0.226, 0.004
Noise	0.092, 0.004	0.019 *, **0.008**	0.344, 0.001	0.130, 0.003	0.159, 0.003
Opposition	0.606, 0.000	0.694, 0.000	0.826, 0.000	0.210, 0.002	0.953, 0.000
Position × Noise	0.814, 0.001	0.715, 0.001	0.929, 0.000	0.603, 0.001	0.194, 0.005
Position × Opposition	0.419, 0.002	0.368, 0.003	0.520, 0.002	0.823, 0.001	0.996, 0.000

Legend: *p*—significance, η^2^*p*—partial eta squared. * *p* < 0.05.

**Table 3:** Spearman correlations between jump and BR variables and shooting accuracy (ordinal scale 0–4). Significance level of *p* < 0.05 *.

Variables	Correlation Analysis			
	Spearman (ρ)	Correlation	*p*	Tendency
Flight Time-Accuracy	−0.081	weak negative	0.001 *	inverse (slightly)
Jump Height-Accuracy	−0.072	weak negative	0.005 *	inverse (slightly)
BR Height-Accuracy	0.116	weak positive	0.002 *	direct (slightly)
BR Angle-Accuracy	0.037	-	0.329	-
BR Velocity-Accuracy	0.084	weak positive	0.025 *	direct (slightly)

## Data Availability

The original contributions presented in this study are included in the article. Further inquiries can be directed to the corresponding author.
